# New insights into the pathogenesis of non-allergic rhinitis

**DOI:** 10.1186/2045-7022-5-S4-P1

**Published:** 2015-06-26

**Authors:** Christine Segboer, Wytske Fokkens, Kees Drunen van

**Affiliations:** 1 Academic Medical Centre Amsterdam, Amsterdam, Netherlands; 2 Academic Medical Centre Amsterdam, Otolaryngology, Amsterdam, Netherlands

## Background

Effective treatment requires a correct diagnosis, however for different subgroups of chronic rhinitis patients differentiation on a clinical level is complicated. In the case of non-allergic rhinitis (NAR) that represents a wide spectrum of diseases, we are also faced with a missing mechanistic or molecular diagnosis. Here we present an unbiased transcriptomic approach on nasal epithelial cells that assess expression differences between chronic rhinitis subgroups.

## Method

Epithelial cells were obtained from concha inferior biopsies of allergic rhinitis (AR), non-allergic rhinitis (NAR) and healthy controls. Affimetric arrays and were used to determine the expression profiles.

## Results

Comparing subgroups of NAR patients with healthy controls three significant differences were observed related to the activation state of genes. Two classes of genes were significantly up-regulated in non-allergic rhinitis. The first class is related to interferon (INF) signalling and the second class is related to ‘epithelial growth factor receptor’ (EGFR) signalling pathway. Finally there was a significant difference in cold-induced RNA binding protein (CIRBP) that was activated in NAR and de-activated in healthy. These three path could well be related as CIRBP is able to affect ERK activity downstream of EGFR resulting in increased IFN-levels.

## Conclusion

Assessing differences between chronic rhinitis subgroups using a completely unbiased approach has revealed a novel profile in non-allergic rhinitis. This profile seems linked to the way nasal airway epithelial cells respond to cold air. The last observation is very relevant because cold-dry air provocation is the golden standard to assess nasal hyperreactivity. The increased activity of the EGFR and INF-pathways in turn might well explain the symptomatology in these patients.

